# Behavioural change interventions encouraging clinicians to reduce carbon emissions in clinical activity: a systematic review

**DOI:** 10.1186/s12913-023-09370-2

**Published:** 2023-04-20

**Authors:** Carys Batcup, Matilde Breth-Petersen, Thomas Dakin, Alexandra Barratt, Forbes McGain, Ben R. Newell, Kristen Pickles

**Affiliations:** 1 Faculty of Medicine and Health, Sydney Health Literacy Lab, School of Public Health, The University of Sydney, Edward Ford Building, A27 Fisher Rd, Sydney, Australia; 2 Western Health, Department of Critical Care Medicine, University of Melbourne, Melbourne, Australia; 3 School of Psychology, University of New South Wales, Sydney, Australia

**Keywords:** Behaviour change, Carbon emissions, Intervention, Healthcare delivery, Clinical activity, Clinicians, Behaviour change wheel, BCT taxonomy, Greenhouse gas emissions, Health system sustainability

## Abstract

**Background:**

Clinical activity accounts for 70–80% of the carbon footprint of healthcare. A critical component of reducing emissions is shifting clinical behaviour towards reducing, avoiding, or replacing carbon-intensive healthcare. The objective of this systematic review was to find, map and assess behaviour change interventions that have been implemented in healthcare settings to encourage clinicians to reduce greenhouse gas emissions from their clinical activity.

**Methods:**

Studies eligible for inclusion were those reporting on a behaviour change intervention to reduce carbon emissions via changes in healthcare workplace behaviour. Six databases were searched in November 2021 (updated February 2022). A pre-determined template was used to extract data from the studies, and risk of bias was assessed. The behaviour change techniques (BCTs) used in the interventions were coded using the BCT Taxonomy.

**Results:**

Six full-text studies were included in this review, and 14 conference abstracts. All studies used a before-after intervention design. The majority were UK studies (*n* = 15), followed by US (*n* = 3) and Australia (*n* = 2). Of the full-text studies, four focused on reducing the emissions associated with anaesthesia, and two aimed at reducing unnecessary test ordering. Of the conference abstracts, 13 focused on anaesthetic gas usage, and one on respiratory inhalers. The most common BCTs used were social support, salience of consequences, restructuring the physical environment, prompts and cues, feedback on outcome of behaviour, and information about environmental consequences. All studies reported success of their interventions in reducing carbon emissions, prescribing, ordering, and financial costs; however, only two studies reported the magnitude and significance of their intervention’s success. All studies scored at least one item as unclear or at risk of bias.

**Conclusion:**

Most interventions to date have targeted anaesthesia or pathology test ordering in hospital settings. Due to the diverse study outcomes and consequent inability to pool the results, this review is descriptive only, limiting our ability to conclude the effectiveness of interventions. Multiple BCTs were used in each study but these were not compared, evaluated, or used systematically. All studies lacked rigour in study design and measurement of outcomes.

**Review registration:**

The study was registered on Prospero (ID number CRD42021272526) (Breth-Petersen et al., Prospero 2021: CRD42021272526).

**Supplementary Information:**

The online version contains supplementary material available at 10.1186/s12913-023-09370-2.

Cutting carbon emissions as a first step toward reducing greenhouse gas (GHG) levels in the atmosphere is the most urgent goal in the climate emergency. Climate change is the ‘single biggest health threat facing humanity’ and is already negatively impacting public health and health systems globally [1]. Paradoxically, the global health sector – hospitals, health services, and its medical supply chain – is responsible for around 5% of global net carbon emissions [2–4], thus inadvertently contributing to irreversible environmental changes and threatening human health and future generations. This carbon footprint is equivalent to the total CO_2_ emissions of entire countries such as India (7.1%) and Russia (4.7%) [5]. To fulfil international commitments to the Paris Climate Change Agreement and decarbonisation of healthcare systems around the world [6], the health sector must take a lead role in reaching net-zero emissions [1].

Carbon footprint modelling has been applied to health systems internationally to quantify the environmental impacts of healthcare services and activities [7]. This has helped determine the areas in which the most carbon emissions are produced and identify priority areas for strategic intervention [7]. For example, 62% of total health service emissions in the UK were from the supply chain, with 24% (of the total) from the direct delivery of care (e.g. on-site fossil fuel use, anaesthetics, inhalers). Life cycle assessments have identified emissions involved in different hospital departments or operations [8–10] that could be reduced simply and cheaply through, for example, reducing waste [11, 12], changing anaesthetic gases used [13], and reducing unnecessary testing [14] and imaging [15].

Since clinical activity itself accounts for 70–80% of the total carbon footprint of healthcare (not buildings, water, and waste) [16, 17] optimising how clinical care is delivered is a key component to decarbonising the health sector. Therefore, a crucial component to reducing the emissions of clinical care is individual behaviour change [18]. Encouraging a shift in clinical behaviour to avoid or replace carbon-intensive healthcare could result in significant health, carbon, and cost savings for the health system. For example, an existing NHS initiative that used prompt cards to ‘nudge’ anaesthetists away from using a potent anaesthetic agent (desflurane) and towards a lower carbon alternative (e.g., sevoflurane) resulted in the equivalent of 30,000 kg less CO_2_ per month for the hospital [19]. A Trainee-Led Research and Audit in Anaesthesia (TRA2SH) group have campaigned for hospitals in Australia and New Zealand to pledge to #DitchTheDes and remove desflurane from their formularies by 2025 (or sooner) [20]. In Australia, unnecessary Vitamin D testing (> 3 million tests per year) cost Medicare more than $87 million in 2020 and a carbon burden equivalent to 28,000–42,000 kg CO_2_e or driving approximately 160,000–230,000 km in a standard, petrol-fueled, passenger car, yet provided no net health benefit for patients [21]. Lastly, pathology and diagnostic imaging account for approximately 9% of healthcare’s carbon footprint in Australia [22]. Opportunities for intervening in this context include turning off scanners to reduce emissions from standby power and reducing ordering of unnecessary imaging or substituting high-impact imaging (e.g., MRI and CT) with lower-impact imaging (e.g., X-Ray and ultrasound) to reduce carbon and costs [15].

Behaviour change interventions are ‘coordinated sets of activities designed to change specified behaviour patterns’ [23]. Systematic reviews of behavioural interventions to reduce carbon emissions in office workplaces [24] and residential buildings [25] have shown that incentives given to individuals (both financial and non-financial) can be very successful, as well as interventions which change the physical environment in some way (such as fitting technologies) and social influences. However, these are lacking in the healthcare sector. It is widely recognised that interventions targeting clinicians are the most effective when implementing changes in the health setting [26], and that behaviour change interventions have demonstrated effectiveness in multiple areas of healthcare [27–29]. These types of interventions aim to change individual clinicians’ behaviour through a variety of methods. One framework for designing behaviour change interventions is the Behaviour Change Wheel (BCW) [23, 30] which has been used extensively in this setting [31, 32]. It characterises interventions and policies aiming to change behaviour and categorises barriers and facilitators to a particular behaviour change into three areas: capability, motivation, and opportunity (COM-B).

Despite the recent emergence of multiple studies estimating the carbon footprint of clinical activity and suggesting emissions reduction strategies via behaviour change [22, 33, 34], no reviews, to our knowledge, have explored the effectiveness of implemented behavioural change interventions, targeting clinicians, to reduce carbon emissions in health settings. To design and implement effective carbon reduction interventions in clinical care in the future, it is essential to identify and understand the nature and scope of existing initiatives internationally and the impact of those interventions have had on healthcare emissions.

This systematic review sought to answer the question: ‘What behaviour change interventions have been implemented in healthcare settings to encourage clinicians to reduce greenhouse gas emissions from their clinical activity?’ We will achieve this by identifying and synthesising global empirical evidence on behavioural change interventions implemented to reduce carbon emissions arising from clinical care provision. The findings of this review will inform the design and development of emissions-reduction interventions in healthcare settings – and ultimately support a shift towards more sustainable healthcare at this critical time for the environment, the future medical workforce, and the global population.

## Methods

The study was registered on Prospero (ID number CRD42021272526) [35]. Study procedures are reported according to the Preferred Reporting Items for Systematic Reviews and Meta-Analyses (PRISMA) guidance [36].

### Types of studies

See Table 1 for inclusion and exclusion criteria. Eligible study types included randomised and non-randomised controlled trials, controlled or uncontrolled before-and-after studies, case series, case studies, and audit. Conference proceedings were excluded initially before being re-introduced due to a limited number of full-text papers identified. No language restrictions were applied.

**Table 1:** Inclusion and exclusion criteria

**Inclusion**	**Exclusion**
• RCTs, before-and-after studies, case series, case studies, audit • Full text published papers explicitly describing interventions available to change the behaviour of individual clinicians towards environmentally sustainable healthcare choices which reduce emissions • No limits on type of behaviour change intervention/s (e.g. audit and feedback, provider education, incentives, reminders) • No limits on type of clinical activity targeted by intervention (e.g. anaesthesia, prescribing, imaging) • Interventions delivered in primary care clinics, hospitals, health clinics, allied health centres, or online. Studies can report on interventions in any country	• Full-text studies describing interventions targeting supply chains/procurement (beyond individual clinician behavioural change) or interventions to reduce emissions outside of clinical activity in the healthcare setting • Studies not describing interventions • Waste/recycling interventions (e.g. improving waste segregation, introducing recycling scheme) • Animal/veterinary studies • Letters, editorials, reviews or commentaries, opinion pieces, protocols

### Types of interventions

Studies were included if they reported on a behaviour change intervention/s implemented in any clinical setting aimed at decreasing greenhouse gas emissions through changing the behaviour (clinical activity) of individual clinicians at their workplace. Interventions could have been initiated by clinicians or healthcare services and implemented in any healthcare setting including primary care clinics, hospitals, health clinics, allied health centres, or online.

Studies were excluded if they described interventions targeting supply chains/procurement (beyond individual clinician behavioural change) or interventions to reduce emissions outside of clinical activity in the healthcare setting. Studies that evaluated interventions designed primarily to address waste/recycling or water and/or energy use were also excluded. Comparisons of the intervention versus no intervention (usual practice) or another intervention were acceptable.

### Outcomes

Our primary outcome was environmental impact (specifically, greenhouse gas emissions/carbon footprint) of clinical activity, measured or modelled directly or indirectly (e.g. estimated from costs, waste and/or energy consumption). Secondary outcomes included financial costs and change in clinical activity (e.g. reduction in anaesthetic gas use or pathology test ordering).

### Search strategy

Six databases were searched (Medline (via OvidSP), Scopus, EMBASE (via OvidSP), Cinahl, Web of Science (Core Collection), and ABI-Inform) for all studies up to November 4, 2021. The search was repeated to update the results on February 7, 2022. The search terms were based on a previous similar review [24], with additional terms based on known relevant papers and librarian suggestions [from the Faculty of Medicine and Health at the University of Sydney]. The complete list of search terms is included in Table 2 below, and the search strategy is shown in Additional file 1: Appendix 1. The terms were searched within article title, abstract, and keywords. Once the final selection was complete, references and citations of full-text papers were also searched, and potentially relevant articles were reviewed.

**Table 2:** Search terms used

**Emissions terms**	**Healthcare terms**	**Intervention terms**
Emission/s, environment, greenhouse gas/es, carbon gas/es pollute/pollution, CO2, carbon footprint, environmental impact, climate change, climate friendly, CO2e, carbon dioxide equivalents	Doctor/s, nurse/s, nursing, dentist/s, anaesthetist/s, health professional, surgery/ies, surgical, health provider/staff, hospital provider/staff. Imaging, MRI, computed tomography, radiograph/er, radiologist/s, clinician/s, physician/s, asthma, inhaler, pathology	Intervention/s, behaviour/s, strategy/ies, messaging, education, program/s, training, nudge/ing, choice/s, implementation, decision support, e-nudge, audit, feedback, incentive/s, communication

### Study selection

We downloaded references identified in searches and uploaded them to Covidence, an online software platform and primary screening and data extraction tool. Two reviewers (CB, MBP) independently conducted title and abstract screening using the inclusion and exclusion criteria to determine suitability. Any conflicts were resolved by discussion with two additional reviewers (KP and TD). All reviewers assessed the full text of the remaining papers.

Once this process was completed, a small number (*n* = 6) of studies were found to be relevant to the review. After a discussion amongst the reviewers, it was decided that we would also include conference abstracts of relevant studies that may be published in the future because of the limited pool of studies. Attempts were made to contact authors of all included conference abstracts to confirm that the study had not (yet) been published.

### Data extraction

Three reviewers (MP, TD, CB) used a pre-determined data extraction template on Excel to extract data from the included full-text papers and conference abstracts. The data extracted from the included studies were: year, country, study design, study population, research question/aim, description of the intervention, measured outcomes (change in CO_**2**_e, change in clinical activity e.g. anaesthetic gas usage, change in cost), and behaviour change techniques used.

**Fig. 1:**
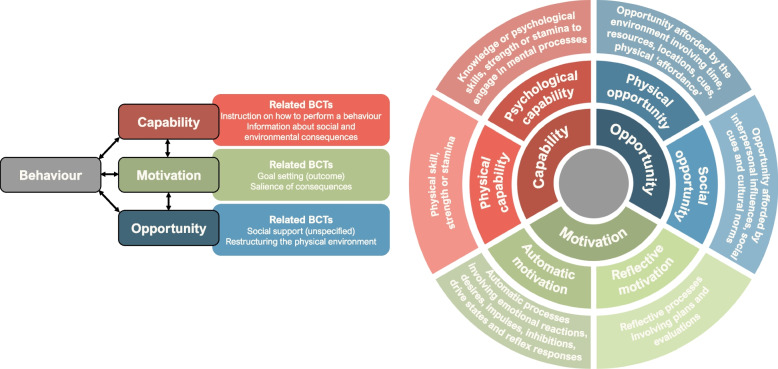
Part of the Behaviour Change Wheel, Capability-Opportunity-Motivation-Behaviour Model (COM-B) definitions and example Behaviour Change Techniques (Figure developed by the research team, using information from the Behaviour Change Wheel and the Behaviour Change Techniques Taxonomy [23, 37]

One reviewer (CB), with training in applying Michie et al.’s behaviour change technique taxonomy [37] to published methods, extracted the behaviour change techniques from the methods section of the full-text papers and as much as possible from methods described in the conference abstracts. We used this taxonomy as it relates to a model of behaviour change commonly used when designing interventions, the Capability-Opportunity-Motivation-Behaviour (COM-B) model [23]. Figure 1 demonstrates how some of the behaviour change techniques, from the taxonomy of 93 techniques, are situated in the COM-B model.

### Assessing the quality of the included studies

For the full-text papers, we used a risk of bias tool for single-arm observational studies of interventions using a modified checklist based on previously-published checklists and evidence-based medicine criteria [38, 39], adapted from [40] (see Additional file 1: Appendix 4 for the tool used). Two reviewers independently rated each study for risk of bias (CB, MBP) and conflicts that arose were discussed with two further reviewers (KP, TD) until consensus was reached. The Template for Intervention Description and Replication (TIDieR) checklist [41] was completed for each study (TD, CB).

### Data synthesis

We anticipated that meta-analysis to calculate the pooled effects of the interventions would not be possible because of heterogenous data reported across the studies and measurement of outcomes. Study findings were therefore synthesised thematically in tabular form.

## Results

### Description of studies

#### Search results

**Fig. 2:**
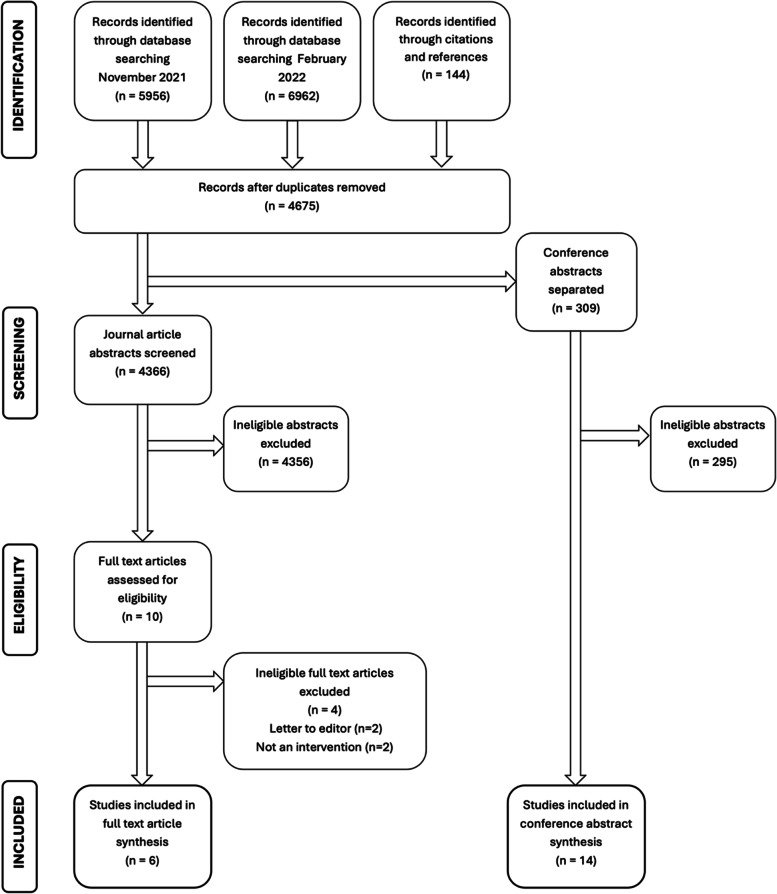
PRISMA diagram

The PRISMA diagram (Fig. 2) shows the search process and results. In November 2021, 5,956 database results were assessed for eligibility. In February 2022, the search was repeated, and an additional 1,006 database results were assessed. The 144 citations and references from the full-text papers included were also reviewed. We identified a total of 4,675 papers (after duplicates were removed). After excluding irrelevant papers based on abstract screening, the full texts of 10 studies were assessed, with six eligible studies identified. Of 309 conference abstracts identified in the search, 14 were found to be eligible for inclusion. The study team emailed lead authors from the conference abstracts to request whether they had progressed to full-text papers. 3 out of the 14 replied and confirmed that they had not. Study details and outcomes are summarised in Tables 3, 4, 5, 6 and 7.

**Table 3:** Characteristics of included studies (*n* = 6)

**Author, Year of publication**	**Study country, setting & target participants**	**Research question/aim**	**Study design**	**Description of behavioural intervention**	**Change in CO**_**2**_**e**		**Other outcomes**	
					**Reduction in amount of CO**_**2**_**e**	**Percentage reduction**	**Change in cost**	**Change in gas usage/test ordering**
Epstein et al., 2016 [42]	US Hospital department Anaesthetists	Does converting to a nonreactive CO_2_ absorbent (1) reduce intraoperative Sevoflurane fresh gas flow (2) reduce Sevoflurane consumption (3) change cost (4) reduce wastage?	Uncontrolled before and after 8 4-week periods before and the same after the intervention; intervention in May 2014	– Change in policy – Regular email updates – Public announcements – Personalised feedback – Canisters changed – Reminder notes on machines **Number of BCTs used: 7**	Not reported	Not reported	Decrease (not significant) of mean cost of Sevoflurane and absorbent purchases of − $293 (*N* = 10 of 10, 95% CI, − $2853 to $2266; *P* = 0.81)	Intraoperative FGF reduced for cases in which Sevoflurane was administered by 435 mL/min (*N* = 8 of 8 periods; 95% CI, 391 to 479; *P* < .0001) Sevoflurane consumption per minute of administration decreased by 0.039 mL/min (*N* = 8 of 8; 95% CI, 0.029 to 0.049 mL/min; *P* < .0001) Decrease (not significant) in average number of bottles of Sevoflurane purchased, from 180.8 ± 6.3 to 160.7 ± 8.5 (*N* = 10 of 10, difference = − 17.1; 95% CI, − 17.4 to 16.8; *P* = .07)
Regan et al., 2018 [43]	UK Paediatric cardiology ward (Bear Ward) in Great Ormond Street Hospital, London Clinical staff on the ward	To investigate whether an educational intervention can reduce combined biochemical tests ordered, and measure financial and carbon dioxide savings	Uncontrolled before and after 8-week study period, baseline data collected October 2014	– Target set – Progress reports – Educational poster – Cartoon-based stickers – Incentivised celebratory tea trolley **Number of BCTs used: 10**	10,042 kgCO_2_e reduction per year	Not reported	Total monthly biochemistry cost for the ward fell from an average of £2,275 to £1,742, equating to a saving of £6,396 in the first year	Increase of C005 tests ordered as a % of total biochemistry tests from 13% to 45% Reciprocal significant reduction in the % of unnecessary combined biochemistry tests ordered
Carter et al., 2019 [44]	UK Royal Free Hospital (RFH) anaesthetic department, North London (teaching hospital) Anaesthetists (10–13 different theatres)	To make anaesthetic practice more environmentally friendly and reduce departmental spending, by promoting use of low-flow anaesthesia and encouraging isoflurane use	Uncontrolled before and after September 2016–March 2017	– Weekly team feedback – Staff presentations – Regular email updates – Made isoflurane vaporisers available – Removed Sevoflurane vaporisers – Poster of progression – Monthly spot audits **Number of BCTs used: 9**	Not reported	Not reported	25% decrease in total expenditure on volatile agents	18% reduction in volatile gas bottles ordered compared with the same period the previous year
Zuegge et al., 2019 [45]	US Hospital, Wisconsin Perioperative setting Anaesthetists	To mitigate the negative environmental and financial impacts of anaesthetic practice in the perioperative setting through multidisciplinary staff engagement and provider education on flow rate reduction and volatile agent choice	Uncontrolled before and after Annual data for 2012 vs annual data for 2015	– Education: lectures and presentations – Regular email updates – Labels added to machines – Community support **Number of BCTs used: 7**	105 kg reduction per case per year 2,865,430 kgCO_2_e reduction per annum	64% reduction in emissions per case, per fiscal year	Savings of $25,000 per month	55% reduction in Desflurane usage (total Desflurane purchasing dropped from 3,025 kg (2012) to 1,354 kg (2015) 16% increase in Sevoflurane usage (from 262 to 305 bottles a month)
Glenski & Levine 2020 [46]	US Children’s Mercy Hospital, Kansas City, Missouri (tertiary paediatric institution) 33 anaesthetists	To decrease Sevoflurane use per anaesthetic using new technology and staff education and encouragement	Uncontrolled before and after Implemented over 9 months	– New machines – Spot checks – Weekly team feedback – Education: demonstration and presentations – New defaults set **Number of BCTs used: 10**	28.5 MTCO_2_e reduction/ year compared to baseline	25% reduction in MTCO_2_e	Not reported	20% decrease in no. of Sevoflurane bottles used/month 25% decrease in the average amount of Sevoflurane used per anaesthetic performed
McAlister et al., 2021 [47]	Australia St George Hospital, Division of Medicine, Sydney	To measure difference in number of pathology collections between an intervention and reference period, and corresponding difference in GHG emissions, financial costs, and adverse events	Retrospective cohort study September 2019–February 2020	– Policy change – Educational posters – Department meetings – Staff orientations **Number of BCTs used: 5**	Reduction of 53 g CO_2_e (95% CI, 24-83 g; *P* < .001) per admission 132 kg CO_2_e saved during intervention period	22% CO_2_e reduction in total	Fees per admission $22 lower (95% CI, $9-$34; *P* = .001)	10% reduction in rate of pathology collections (95% CI, 0.86–0.95; *P* < .001)

**Table 4:** Characteristics of conference abstracts (*n* = 14)

**Author, Year of publication**	**Study country, setting & target participants**	**Research question/aim**	**Design**	**Description of intervention**	**Change in CO**_**2**_**e**		**Other outcomes**	
					**Reduction in amount of CO**_**2**_**e**	**Percentage reduction**	**Change in cost**	**Change in gas usage/test ordering**
Patel et al., 2014 [48]	US Anaesthesia providers	Can a personalised email feedback system result in a reduction in fresh gas flow during general anaesthesia?	Uncontrolled before and after (QIP) July–October 2013	– Target set – Personalised feedback – Regular email updates **Number of BCTs used**^**a**^**: 5**	Not reported	Not reported	Not reported	FGF reduction of: - 0.34 (± 0.03) l/min for Desflurane - 0.39 (± 0.02) l/min for Sevoflurane
Boyle et al., 2018 [49]	UK (Scotland) Hospital operating theatres *N* = 45 anaesthetists and theatre staff	Can small changes in the working environment reduce the use of volatile agents?	Uncontrolled before and after September 2015–March 2018	– Purchasing of new anaesthetic machines – Staff education – Made Sevoflurane and isoflurane default agents; Desflurane available on request only **Number of BCTs used**^**a**^**: 4**	320122 kg CO2e reduction over a 6-month period (comparing Oct-March 2016 and Oct-March 2018)	81% reduction over this period	52% saving, from £64,606 in April-Sept 2016 to £30,9423 in Oct-March 2018	Not reported
Danby et al., 2018 [50]	UK (England) The Feeman Hospital, Newcastle Hospitals NHS Trust Operating theatre department (*n* = 17 general theatres)	To measure the carbon dioxide equivalency of anaesthetic agent usage and encourage a more sustainable approach via altering volatile agent choice and informing staff	Uncontrolled before and after Follow-up 3 months	– Gas use converted into everyday equivalents – Staff presented with information on environmental impact of volatile agents **Number of BCTs used**^**a**^**: 4**	13 CO_2_e kg/hr^−1^ reduction	Not reported	Not reported	Not reported
Jani & Kalla, 2018 [51]	UK (England) Milton Keynes University Hospital NHS Trust Operating theatre department (*n* = 54)	Can audit and reminders encourage staff to switch off anaesthetic gases at time of transfer and use low-flow anaesthesia technique during anaesthesia	Uncontrolled before and after Follow-up 6 months	– Findings of baseline survey/audit shared with staff – Visual prompts on theatre doors and anaesthetic machines – Email reminders **Number of BCTs used**^**a**^**: 5**	Not reported	Not reported	Not reported	Marked improvement in anaesthetic gases being switched off at the time of transfer (not quantified)
Hickman & Molyneux, 2019 [52]	UK (England) University Hospitals Bristol (UHB) NHS Foundation Trust Anaesthetic staff	To reduce total CO2e produced from vaporisation of Desflurane through implementation of an interactive change management programme	Uncontrolled before and after Data analysed 6 months pre- and post-intervention	– Volatile gas usage converted into tangible everyday equivalencies – Educational prompt cards placed on top of all anaesthetic machines – Prizes awarded **Number of BCTs used**^**a**^**: 6**	17,087 kgCO_2_e total reduction, comparing the 6 months pre and post intervention	28% reduction	Not reported	Desflurane reduction of 119,610 kg
Lawson & Baxter, 2019 [53]	UK (England) Newcastle-upon-Tyne hospitals 19 operating theatre departments	To determine whether a reduction in Desflurane use is possible, using a behavioural nudge	Uncontrolled before and after (QIP) 2019 – data analysed 5 days pre- and post-intervention	– Desflurane removed from machines and vaporiser setup was standardised to sevoflurane and isoflurane on every machine – Changed default vaporiser location **Number of BCTs used**^**a**^**: 2**	1,499 kgCO_2_e reduction	9% reduction pre vs post intervention	£17.56 cost saving	Desflurane use was reduced post-intervention in favour of increased sevoflurane usage (not quantified)
Self & Eveleigh, 2019 [54]	UK (England) Gloucestershire Hospitals NHS Foundation Trust Anaesthetic department	To change practice at the Trust to reduce Desflurane use	Uncontrolled before and after Measurement for 6 months post-intervention (2018), compared to the same period the year before (2017)	– Educational presentation **Number of BCTs used**^**a**^**: 3**	800 tonnes CO_2_e per annum	Not reported	£70,000 cost saving per annum	69% reduction in Desflurane use in the 6 month period post intervention vs pre
Benness & Doane, 2021 [55]	Australia North Shore Hospital, Sydney	To promote clinically appropriate, socially responsible usage of volatile anaesthetics and assess the impact on carbon footprint	Follow up audit at 9 months	– Educational presentation – Departmental newsletters – Educational posters **Number of BCTs used**^**a**^**: 5**	360 tonne CO_2_e reduction over the 9 months since the intervention	Not reported	> $46,000 cost savings	> 58% reduction in Desflurane use
Carta et al., 2021 [56]	UK (England) Salisbury District Hospital Anaesthetists	Can staff encouragement reduce use of volatile anaesthetics?	Uncontrolled before and after Baseline survey carried out over a 2-week period in Oct 2019; presented to staff June 2020	– Findings of baseline audit presented to staff, with encouragement to use lower-carbon volatile agents – Follow-up email – Reminder stickers placed on all machines **Number of BCTs used**^**a**^**: 7**	Not reported	74% reduction of CO_2_e per hour	Not reported	Use of Sevoflurane increased from 49 to 98% Use of nitrous oxide fell from 34 to 28% The average flow rate fell from 2.37 to1.9 l/min
Hirst et al., 2021 [57]	UK (England) East Lancashire Hospitals NHS Trust Anaesthetics department	To reduce Desflurane usage in theatres using a simple intervention	Uncontrolled before and after Baseline measurements – Feb 2017–18 Intervention—January 2020 Post-intervention measurements – Feb 2020–21	– Desflurane vaporisers were removed from anaesthetic machines **Number of BCTs used**^**a**^**: 2**	4,563 tonnes CO_2_e reduction	84%	£100,390 cost reduction	Not reported
Jameson & Young, 2021 [58]	UK (Scotland) Glasgow Royal Infirmary and Stobhill Hospital General anaesthetic trainees	Can a quality improvement project achieve a reduction of fresh gas flow rates	Uncontrolled before and after	– Target set – Educational presentation to trainees – Email reminders **Number of BCTs used**^**a**^**: 4**	Not reported	Not reported	Not reported	Cases with induction of flow rate of ≤ 6 l/min increased from 11% to 38.5% (*p* = .0001) Cases with LFA increased from 40 to 45% (*p*= .54460)
Kirkman et al., 2021 [59]	UK (England) Doncaster Royal Infirmary Anaesthesia staff	To measure change in volatile usage following removal of Desflurane as a default vaporiser and staff education	Uncontrolled before and after	– Desflurane removed as default vaporiser on all machines – Educational posters – Monthly presentations **Number of BCTs used**^**a**^**: 4**	40 kgCO_2_e reduction per theatre case 34,840 kgCO_2_e reduction per month	70% reduction in CO2e per case from Sept 2020 to Feb 2021	₤1,200 per month cost saving	27% reduction in Desflurane use Reciprocal increase in Sevoflurane use
Roome et al., 2021 [60]	UK (England)	To offer and promote safe inhaler disposal and a recycling scheme, and empower staff and patients to make sustainable inhaler decisions at an individual level	Uncontrolled before and after	– Created a decision support tool for GPs to use inhalers with lower carbon footprints – Prompts when prescribing inhalers – Summary of greener inhalers given to GPs **Number of BCTs used**^**a**^**: 3**	Savings of 363 tonnes of CO_2_e per year	Not reported	Not reported	Reduced number of Ventolin inhalers being prescribed by 31%
Wilson & Clark, 2021 [61]	UK (Scotland) University Hospital Crosshouse, NHS Surgical department, and individual anaesthetists	Can presenting usage data to staff lead to a reduction in volatile agent usage	Uncontrolled before and after Usage data collected 4 years prior to 2018 intervention and 20 months after (compared 2016 and 2019)	– Audit mean measurements presented to the department – Individual audit revealed privately to anaesthetists **Number of BCTs used**^**a**^**: 2**	Not reported	Not reported	Cost reduction per month of £1,517 for Desflurane and £,1067 for Sevoflurane, an annual saving of £31,000	46.9% reduction in Desflurane use 26.2% reduction in Sevoflurane use

^a^Estimate based on what was reported in the abstract

**Table 5:** Behaviour change techniques (BCTs) for full text papers

Author, year	Behaviour Change Techniques													Total number of BCTs used
	1.3	1.6	2.1	2.7	3.1	4.1	5.2	5.3	7.1	9.1	10.8	12.1	12.5	
	Goal setting (outcome)	Discrepancy between current behaviour and goal	Monitoring of behaviour by others without feedback	Feedback on outcome(s) of behaviour	Social support, unspecified	Instruction on how to perform a behaviour	Salience of consequences	Information about social and environmental consequences	Prompts / cues	Credible source	Incentive (outcome)	Restructuring the physical environment	Adding objects to the environment	
Epstein et al., 2016 [42]		✔		✔		✔			✔	✔		✔	✔	7
Regan et al., 2018 [43]	✔			✔	✔	✔	✔	✔	✔	✔	✔		✔	10
Carter et al., 2019 [44]				✔	✔	✔	✔	✔	✔	✔		✔	✔	9
Zuegge et al., 2019 [45]					✔	✔	✔	✔	✔	✔			✔	7
Glenski et al., 2020 [46]		✔	✔	✔	✔	✔	✔	✔	✔	✔		✔		10
McAlister et al., 2021 [47]					✔	✔	✔			✔			✔	5

Number corresponds to the code in the Behaviour Change Technique Taxonomy [30]

**Table 6:** Behaviour change techniques (BCTs) for conference abstracts

Author, year	Behaviour Change Techniques								
	1.3	2.7	4.1	5.2	5.3	7.1	9.1	12.1	12.5
	Goal setting (outcome)	Feedback on outcome(s) of behaviour	Instruction on how to perform a behaviour	Salience of consequences	Information about social and environmental consequences	Prompts / cues	Credible source	Restructuring the physical environment	Adding objects to the environment
Patel et al., 2014 [48]	✔	✔			✔	✔	✔		
Boyle et al., 2018 [49]					✔		✔	✔	✔
Danby et al., 2018 [50]		✔		✔	✔		✔		
Jani & Kalla, 2018 [51]		✔	✔			✔	✔		✔
Hickman & Molyneux, 2019 [52]			✔	✔	✔	✔	✔		✔
Lawson & Baxter, 2019 [53]							✔	✔	
Self & Eveleigh, 2019 [54]			✔		✔		✔		
Benness & Doane, 2021 [55]			✔		✔	✔	✔		✔
Carta et al., 2021 [56]		✔	✔	✔	✔	✔	✔		✔
Hirst et al., 2021 [57]							✔	✔	
Jameson & Young, 2021 [58]	✔		✔			✔	✔		
Kirkman et al., 2021 [59]					✔		✔	✔	✔
Roome et al., 2021 [60]			✔			✔	✔		
Wilson & Clark, 2021 [61]		✔					✔		

Number corresponds to the code in the Behaviour Change Technique Taxonomy [32]

**Table 7:** Behaviour change techniques (BCTs) definitions from the taxonomy from Michie et al.

**Code in the BCT Taxonomy**	**BCT**	**Definition from the BCT Taxonomy**	**Example from the papers**
1.3	Goal setting (outcome)	Set or agree on a goal defined in terms of a positive outcome of wanted behaviour	Target set of Desflurane reduction
1.6	Discrepancy between current behaviour and goal	Draw attention to discrepancies between a person’s current behaviour (in terms of the form, frequency, duration, or intensity of that behaviour) and the person’s previously set outcome goals, behavioural goals or action plans (goes beyond self-monitoring of behaviour)	“The low flow wizard displays the required FGF and the user’s current flows”
2.1	Monitoring of behaviour by others without feedback	Observe or record behaviour with the person’s knowledge as part of a behaviour change strategy	“Confirmation rounds were performed on a random basis. This involved checking to see whether the provider was utilising the low flow wizard”
2.7	Feedback on outcome(s) of behaviour	Monitor and provide feedback on the outcome of performance of the behaviour	“Each anesthesia provider was emailed a report describing his or her FGF for each agent over the prior 12 months”
3.1	Social support (unspecified)	Advise on, arrange or provide social support (e.g. from friends, relatives, colleagues,’ buddies’ or staff) or noncontingent praise or reward for performance of the behaviour. It includes encouragement and counselling, but only when it is directed at the behaviour	“Efforts were made via outreach relationship building and one-on-one conversations…addressing any concerns as well as empowering local passionate champions “
4.1	Instruction on how to perform a behaviour	Advise or agree on how to perform the behaviour (includes ‘Skills training’)	“Provided a practical guide to selecting the common C005 test required on the computer system”
5.2	Salience of consequences	Use methods specifically designed to emphasise the consequences of performing the behaviour with the aim of making them more memorable (goes beyond informing about consequences)	“A graphic designer was employed to create labels with images intended to elicit an emotional response”
5.3	Information about social and environmental consequences	Provide information (e.g. written, verbal, visual) about social and environmental consequences of performing the behaviour	“The many benefits of low flow anaesthesia including decreased costs and environmental impact were also distributed”
7.1	Prompts / cues	Introduce or define environmental or social stimulus with the purpose of prompting or cueing the behaviour. The prompt or cue would normally occur at the time or place of performance	“Added a notification to change the absorbent to the text message to turn over the operating room.”
9.1	Credible source	Present verbal or visual communication from a credible source in favour of or against the behaviour	“Change in policy was approved and endorsed by the chair and vice chair of the department”
10.8	Incentive (outcome)	Inform that a reward will be delivered if and only if there has been effort and/or progress in achieving the behavioural outcome (includes ‘Positive reinforcement’)	“Incentive of a celebratory tea trolley for staff if the reduction target was met and maintained”
12.1	Restructuring the physical environment	Change, or advise to change the physical environment in order to facilitate performance of the wanted behaviour or create barriers to the unwanted behaviour (other than prompts/cues, rewards and punishments)	“Desflurane vaporisers were removed from anaesthetic machines”
12.5	Adding objects to the environment	Add objects to the environment in order to facilitate performance of the behaviour	“Educational posters were displayed in anaesthetic rooms”

### Full text papers

#### Characteristics of included studies

All studies described interventions in hospital settings. Four of the six included studies were undertaken in hospital anaesthesia departments in the USA [42, 45, 46] (3/6), and UK [44] (1/6). These studies (4/6) focused on reducing emissions of volatile anaesthetic agents, for example by encouraging anaesthetists to use a low flow anaesthesia technique or reducing the use of specific anaesthetic gases – such as desflurane—that have a particularly potent global warming potential. The remaining two studies (2/6) aimed to reduce unnecessary test ordering in a paediatric cardiology ward in the UK and a hospital in Australia (Regan et al. focused on biochemistry [43] and McAlister et al. on pathology [47]).

All included studies used a before-after (pre-post) intervention study design. The main outcome was most commonly measured at baseline for the whole hospital department, followed by an intervention administered to the staff in the department, and the outcome measured again, often many times over a period of months (range of 2 months to 4 years). However, one study [47] measured the effect of the intervention retrospectively, rather than designing the intervention and following its outcomes over time.

The interventions themselves included many different facets. Some common themes were: adding reminders onto machines for which/how much gas to use, sending personalised feedback to individuals based on performance (e.g. how much gas had been used in the last month, and the target), physically changing the canisters in the rooms so that it is more difficult to use the unwanted gas, gathering the team regularly for updates in person, and putting up educational posters. For more detail of the characteristics of included studies’ interventions, the completed TIDieR checklist [41] for each study is in Additional file 1: Appendix 6.

#### Behaviour change techniques (BCTs) used in interventions

The studies in this review used very similar methods to change behaviour. Table 5 shows the BCTs coded from the full-text papers (see Additional file 1: Appendix 2 for more detail, and Table 7 for definitions of the techniques included). Five to 10 BCTs were used in each study; all included papers used the techniques of (1) credible source, as they all took place in a hospital setting and interventions were run by colleagues of participants, and (2) instruction on how to perform a behaviour, such as a guide to using new machines to deliver anaesthesia.

Most of the studies included the BCTs of social support (5/6; e.g. encouraging staff), salience of consequences (5/6; e.g. showing carbon emissions reductions as equivalent miles driven by a car), adding objects to the environment (5/6; e.g. posters put up), prompts or cues (5/6; e.g. reminder labels on machines), feedback on outcome of behaviour (4/6; e.g. by sending email updates on progress), and information about environmental consequences (4/6; e.g. describing environmental impacts of the emissions). For a more detailed table of the BCT coding, see Additional file 1: Appendix 2.

#### Outcomes from the interventions

Table 3 summarises primary and secondary outcomes measured across the studies, which were change in CO_2_e (both percentage and absolute difference), change in cost, and change in clinical activity (e.g., anaesthetic gas use or pathology test ordering). All studies reported success in their interventions; however, only two reported any statistical analysis to measure the size and significance of the effect [42, 47].

In terms of the primary outcome of CO_2_e reduction, four studies calculated a reduction in emissions as a result of their intervention. Zuegge et al. reported a reduction of 2,865,430 kg CO_2_e per year, calculating CO_2_e using the global warming potential (GWP) of the gas and the mass emitted [45]. They also reported a CO_2_e per case of 163 kg before their intervention, versus 58 kg 3 years later, in 2015 [45]. Glenski & Levine calculated a reduction of 28.5 MT CO_2_e per year compared to before their intervention, using a formula based on number of Sevoflurane bottles, their density and the GWP [46]. McAlister et al. reported a reduction of 53 g CO_2_e (*P* < 0.001) per admission using an analysis which included many factors (e.g., current energy suppliers, differences in power when a test is taken) [47]. Regan et al. [43] estimated a 10,042 kg CO_2_e reduction per year attributable to a reduction in test ordering after their intervention, using estimates from the UK government’s carbon emissions indicator [62], which convert price to CO_2_.

Of those studies that aimed to change anaesthetic gas use [42, 44–46], all four reported a reduction in emissions after the intervention, through for example, increasing uptake of low flow anaesthesia or replacing more potent inhalational anaesthesia with lower emissions alternatives. Epstein et al. [42] reported a statistically significant reduction in Sevoflurane use (*P* < 0.001) and a non-significant decrease in the number of gas bottles purchased (*P* = 0.81), when comparing 8-week periods before and after their intervention. Carter et al. [44] reported an 18% reduction in volatile gas bottles ordered in the year after their intervention compared to the year before; Zuegge et al. [45] a 55% reduction in Desflurane use (and a 16% increase in Sevoflurane use) in the yearly totals before and after their intervention; and Glenski & Levine [46] reported a 20% decrease in Sevoflurane bottles used per month (and a 25% decrease in the amount of Sevoflurane used per anaesthetic performed) before their intervention compared to 9 months later.

Two studies described the effect of interventions that aimed to reduce the cost and environmental impact of unnecessary test ordering. Regan et al. [43] reported a significant reduction in percentage of biochemical tests ordered, as well as an increase in use of more efficient C005 tests as a percentage of total biochemistry tests ordered, from 13 to 45%. McAlister et al. [47] found a 10% reduction in rate of pathology collections (*P* < 0.001) after the intervention.

Five of six studies also found a reduction in financial cost. Epstein et al. [42] reported a non-significant decrease in the cost of absorbent purchases (*P* = 0.81). Carter et al. [44] reported a 25% decrease in spending on volatile agents compared with the same period the previous year. Zuegge et al. [45] calculated savings of $25,000 per month after their intervention. McAlister et al. [47] also found that fees per admission were $22 lower (*P* = 0.001) after their intervention, and for Regan et al. [43] biochemistry test cost fell by £533 (23%) per month after their intervention.

### Conference abstracts

#### Characteristics of included studies

Almost all the studies (13/14) described in the conference abstracts were conducted in the UK, with the remaining one from Australia [55]. Most of the studies (13/14) were focused on anaesthetic gas usage, with only one aiming to reduce emissions of respiratory inhalers [60]. In terms of methodology, all abstracts described before-after (pre-post) study design. One of the studies [60] was aimed at GPs; the rest targeted anaesthetists in hospitals (13/14).

As limited detail was included in the abstracts, interventions were not described as thoroughly as in the full-text papers. However, some common characteristics of the interventions described were: participant feedback on progress via, for example, email updates, visual prompts such as stickers on machines, education provided through presentations and posters, and physically removing the unwanted gas (usually Desflurane) from anaesthetic machines. The one intervention that focused on inhalers was slightly different [60]. Here, GPs were provided with educational materials on the environmental benefits of using less carbon intensive inhalers, as well as a decision support tool to use with patients and prompts when prescribing inhalers.

#### Behaviour change techniques (BCTs) used in interventions

Table 6 includes as many BCTs as possible from the abstracts (see Additional file 1: Appendix 3 for more detail, and Table 7 for definitions of the techniques included). Like the full text papers, the conference abstracts also all included the technique of ‘credible source’. Most also included information about environmental consequences (8/14; e.g., presentation on environmental impacts). Also common were instruction on how to perform a behaviour (7/14; e.g., a website with education and instructions), and prompts or cues (7/14; e.g., reminders on machines). For a more detailed summary, see Additional file 1: Appendix 3.

#### Outcomes from the interventions

Primary and secondary outcomes of the conference abstracts are summarised in Table 4, where detail was available. Only one study reported statistical analyses as a measure of effect [58]. All reported an effect on outcomes following the intervention. Nine of the 13 anaesthetic gas studies found a reduction in gas use, and the study aiming to reduce Ventolin inhaler prescribing [60] achieved a 31% reduction in inhalers being prescribed after their intervention, and improved patient satisfaction. Similar to the full-text papers, 7/14 abstracts also reported a reduction in financial cost and 8/14 a reduction in CO_2_e emissions.

### Quality of the evidence

Risk of bias assessment for the 6 included studies is reported in Table 8; definitions of the risk of bias criteria and detailed rationale per study are provided in Additional file 1: Appendices 4 and 5. Overall, all studies scored at least one item as unclear or at risk of bias.

**Table 8:** Risk of bias for full-text papers

**Paper**	**Internal validity**				**External validity**			
	**Selection bias: representative?**	**Attrition bias: adequate?**	**Detection bias: blind?**	**Confounding: adjustment?**	**Reporting bias (study group): well defined?**	**Reporting bias (follow-up): well defined?**	**Reporting bias (outcome): well defined?**	**Analyses: well defined?**
**Epstein et al., 2016** [42]	✔	✔	**✖**	**?**	**?**	✔	✔	✔
**Regan et al., 2018** [43]	✔	✔	**✖**	**✖**	**?**	✔	✔	**✖**
**Carter et al., 2019** [44]	✔	✔	**✖**	**✖**	**✖**	✔	✔	**✖**
**Zuegge et al., 2019** [45]	✔	✔	**✖**	**✖**	**✖**	✔	✔	✔
**Glenski et al., 2020** [46]	✔	✔	**✖**	**✖**	**?**	✔	✔	✔
**McAlister et al., 2021** [47]	✔	✔	**✖**	**?**	**?**	✔	✔	✔

#### Internal validity

Selection bias was assessed as low risk for all studies, as the total eligible population (e.g., entire hospital department) was included in the intervention (although none explored how many individuals within the departments were engaged with the intervention). Study outcomes were also assessed in > 95% of the study group of interest, meaning that risk of attrition bias was judged to be low for the included studies. All 6 studies were judged to be at high risk of detection bias because all studies did not have blinded outcome assessors (except McAlister et al., however, in this study, they also were not blinded to the investigated determinant [47]). Regarding adjustment for confounding factors, four of the six studies [43–46] did not report adjusting for any confounders. The remaining two did account for some but not fully. McAlister et al. included sensitivity analyses adjusting for age, sex, NWAU19 and type of admission but reported in their limitations that they did not capture all confounding factors due to a lack of control in the study [47]. Epstein et al. conducted a sensitivity analysis on the financial implications of changing gases, however not for any other of their hypotheses [42].

#### External validity

Reporting bias was not well defined for three studies: Carter et al. [44] and Zuegge et al. [45] did not adequately define the number of participants or the intervention. Glenski & Levine defined the number of participants (number of people in the anaesthesiology department) but did not define the intervention adequately enough [46]. Epstein et al. [42], Regan et al. [43] and McAlister et al. defined the intervention but not the number of participants, as they did not include the number of people in the department being targeted by the intervention [47]. All studies defined the follow up and outcome adequately. Despite four of the studies also defining the method of analysis and quantifying the effect of the intervention [42, 45–47], two studies [43, 44] did not perform any kind of rigorous statistical analyses.

## Discussion

### Principal results

Six studies that described behaviour change interventions to reduce greenhouse gas emissions in healthcare settings were identified for inclusion in this review. 14 conference abstracts were also identified that met eligibility criteria. All studies took place in hospitals. The most common techniques included in the behaviour change interventions were: credible source, social support, salience of consequences, adding objects to the environment, and prompts or cues. Four looked at changing or reducing anaesthetic agents, a key carbon emitter, with their interventions resulting in 16–55% reduction in gas used. The other two aimed to reduce unnecessary test ordering to lower emissions; these two interventions were also successful in their aims.

However, not all studies measured or calculated CO_2_e, despite a reduction in emissions being their goal. Those studies that did report a reduction, for example Regan et al.’s calculated reduction of 10,042kg [43], were not necessarily reporting an accurate estimate of carbon emissions saved because their estimate is based on a conversion of cost to carbon emissions. While cost-based estimates of carbon emissions are widely used, they are less accurate than emissions estimate from life cycle assessment for a range of reasons including some components of the life cycle of the product/service may be omitted, and the assumption of a linear relationship between costs and carbon emissions may not always hold. One study [47] used rigorous methodology (i.e., environmental impact was based on previous LCA), however others used simple calculations which may not account for all CO_2_e emissions. Therefore, the quantifiable reductions in number of tests ordered and amount of harmful gases (e.g. desflurane) used may be a better indicator of reduced environmental impact.

### Strengths and limitations

As far as the authors are aware, this review is the first to look at behaviour change interventions to reduce carbon emissions in healthcare settings. It provides a valuable starting point for others to design interventions in similar contexts as it demonstrates the type and scope of behavioural change interventions implemented internationally to address the carbon footprint of clinical care. It shows interventions to date have targeted anaesthetic gas use or unnecessary pathology test ordering in hospital settings only. A strength of this study is our systematic mapping of behavioural change techniques used in each study. Other strengths include the extensive search strategy and large number of titles and abstracts screened and having multiple assessors to extract data and conduct risk of bias assessments independently.

However, only 6 studies were eligible to be included in this review, and eligible conference abstracts (which were included to illustrate some local initiatives that have been undertaken by clinicians in their clinical settings) are unlikely to be published in academic journals in the future. Furthermore, the 6 included studies were very similar in methodology and interventions. None of the studies randomised groups to different interventions or had a control group. The only study design was uncontrolled before-after. In order to conclude that the interventions were indeed effective, we need gold standard RCTs to truly measure their effectiveness. As statistical analyses was not performed in most of the included full-text papers, we cannot be sure of how much of an improvement the interventions caused and whether this was statistically significant.

Another limitation is that the studies introduced new aspects of the intervention as it progressed, with limited or no effort made to evaluate the effects of each component, so it remains unclear which aspect of the intervention produced the largest effect on the outcomes measured. The studies used multiple behavioural change techniques, for example adding objects to the environment and providing tailored feedback, but did not compare any, or use them systematically or strategically. Therefore, it is not clear which techniques were the most effective, or indeed if any were counter-intuitive.

None of the studies appear to have designed their intervention using a model, theory or framework which is recommended when designing successful behaviour change interventions [63, 64]. All included studies did not reference or define the barriers and facilitators involved in the behaviour they were attempting to change. This is best practice when aiming to change behaviour, so as to ensure the intervention is targeting the barriers to behaviour change [23].

### Comparison to prior work

Previous work in other areas has also found that the behaviour change techniques of incentives and social influences work well, as well as changing the environment [24, 25]. This review demonstrates success using social support and adding or removing objects in the environment in the healthcare setting too. However, incentives were only used in one of the studies [43], and this was in combination with 9 other BCTs [43], so it isn’t clear how effective this specific technique is in this setting.

This review shows that very little work has been done to date to develop and evaluate behaviour change interventions to reduce the carbon footprint of healthcare. Yet this area is in urgent need of attention if commitments by countries at the COP26 meeting to move towards low carbon healthcare systems are to be met [65]. Measuring carbon emissions from healthcare is challenging, and methods for doing this work accurately, while well developed in other sectors, are only now being applied to healthcare (e.g., [14, 15, 66, 67]). The gold standard method is the Life Cycle Assessment (LCA), as described by ISO 14040 and ISO 14044. Most of the studies in our systematic review did not measure carbon emissions directly based on LCA, but estimated them, for example from changes in anaesthetic gas usage. This approach is likely to be valid, as in turn those anaesthetic gases have been thoroughly studied in previous LCAs. One study [47] was able to estimate carbon emissions from a previous LCA of common pathology tests. Such studies are currently rare in healthcare. Other studies in our review, which estimated carbon emissions from costs data, are likely less accurate in their estimates of CO_2_e emissions. This highlights the urgent need for LCAs of more healthcare products and services. This work will be needed for better measurement of the outcomes of behavioural interventions to reduce the carbon footprint of healthcare.

### Future research

As most of the interventions of the included studies were designed and run by clinicians themselves, we suggest that there is strong clinician interest and engagement with this issue, which is promising for future research being implemented and maintained in hospitals. However, future research should aim to run these interventions in a systematic and evidence-based way. One method would be to use the COM-B model [23], a tool for designing interventions based on tackling the capability, opportunity and motivational barriers for individuals to perform a particular behaviour. Once the barriers are understood, the relevant BCTs can be applied which target those barriers. Future interventions should also test a select few techniques at a time only, with a control or comparison group, as doing so would give a clearer indication of which specific BCTs are effective, resulting in more practical outcomes that can be implemented in other contexts. High quality evidence is necessary to direct change in clinical behaviour, and close attention must be paid to local contexts (e.g. resources, culture and values, receptivity) for successful implementation of evidence-based interventions into practice, that best support healthcare decarbonisation [33, 68]. Clinicians could be supported to partner with researchers to improve research design, quality, and long-term evaluation.

We also found that most interventions to reduce emissions in healthcare have focused on anaesthesia. This is a priority area due to the high global warming potential of anaesthetic gases – prioritisation which has been made possible by previous LCA studies of these and other greenhouse gases. The behaviour changes observed in these studies represent big wins in terms of large-scale emissions reduction. However, rigorous studies with well-designed interventions are also needed in other areas of clinical practice both in and outside of hospital settings within health care. Future research should investigate what types of interventions could work in other settings, such as primary care or allied health, where there are likely to be different barriers and facilitators to behavioural change.

## Conclusion

To conclude, this review demonstrates that there has been little published research on behaviour change interventions to reduce carbon emissions in healthcare. Those that do exist have all used a before-after design and have tested multiple interventions simultaneously, limiting the reliability of their findings, and have focused on either anaesthetic gas use or test ordering. Future research should be more systematic when designing interventions in this space, and test more rigorously their impact. More interventions should also be done in other areas of healthcare, such as in primary care or other hospital settings.

## Supplementary Information

**Additional file 1: Appendix 1.** Full search strategy. **Appendix 2.** BCTs full codes from papers. **Appendix 3.** BCTs full codes from abstracts. **Appendix 4.** Risk of bias information. **Appendix 5.** Risk of bias more detailed table. **Appendix 6.** TiDIER table.

## Data Availability

The datasets supporting the conclusions of this article are included within this published article and its supplementary information files.

## Abbreviations

BCTs: Behaviour Change Techniques
CO2e: Carbon dioxide equivalent
COM-B model: Capability, Opportunity, Motivation, Behaviour Model
COP26: Conference of the Parties 26
LCA: Life Cycle Assessment
NWAU19: National Weighted Activity Units, 2019
