# Supplementary material for: Behavioural change interventions encouraging clinicians to reduce carbon emissions in clinical activity: a systematic review
Source: BMC Health Serv Res. 2023 Apr 20;23:384. doi: 10.1186/s12913-023-09370-2 (PMC10116654; doi:10.1186/s12913-023-09370-2)
Supplement: Supplementary file 1 — Additional file 1: Appendix 1. Full search strategy. Appendix 2. BCTs full codes from papers. Appendix 3. BCTs full codes from abstracts. Appendix 4. Risk of bias information. Appendix 5. Risk of bias more detailed table. Appendix 6. TiDIER table. [file 12913_2023_9370_MOESM1_ESM.docx]

**Appendices**

**Appendix 1 – Full search strategy**

**Appendix 2 – BCTs full codes from papers**

**Appendix 3 – BCTs full codes from abstracts**

**Appendix 4 – Risk of bias information**

**Appendix 5 – Risk of bias more detailed table**

**Appendix 6 – TiDIER table**

**Appendix 1 – Full search strategy**

| **SCOPUS** |  |  |
| --- | --- | --- |
| (emission* W/10 (environment* OR "greenhouse gas*" OR carbon OR gas OR gases OR pollut* OR CO2)) OR "carbon footprint" OR "greenhouse gas" OR "greenhouse gases" OR (environment* W/1 impact*) OR "climate change" OR "climate friendly" OR "climate-friendly" OR "CO2e" OR "carbon dioxide equivalents" | doctor OR doctors OR nurse OR nursing OR nurses OR dentist* OR an*esthe* OR "health professional*" OR surgery OR surgeries OR surgical OR (health OR hospital W/3 (staff OR provider)) OR (imaging W/3 (medical OR nuclear OR diagnostic OR radiological OR ultrasound)) OR "magnetic resonance imaging" OR MRI OR "computed tomography" OR radiograph* OR radiologist* OR radiographer* OR clinician* OR physician* OR (asthma W/10 inhal*) OR pathology | intervent* OR behavio* OR incentiv* OR strateg* OR messaging OR educat* OR program* OR training OR implement* OR communicat* OR nudg* OR choice* OR e-nudge OR "decision support" OR audit OR feedback |
|  |  |  |
| **OVID** |  |  |
| (emission* ADJ10 (environment* OR "greenhouse gas*" OR carbon OR gas OR gases OR pollut* OR CO2)) OR "carbon footprint" OR "greenhouse gas" OR "greenhouse gases" OR (environment* ADJ impact*) OR "climate change" OR "climate friendly" OR "climate-friendly" OR "CO2e" OR "carbon dioxide equivalents" | doctor OR doctors OR nurse OR nursing OR nurses OR dentist* OR an?esthe* OR "health professional*" OR surgery OR surgeries OR surgical OR (health OR hospital ADJ3 (staff OR provider)) OR (imaging ADJ3 (medical OR nuclear OR diagnostic OR radiological OR ultrasound)) OR "magnetic resonance imaging" OR MRI OR "computed tomography" OR radiograph* OR radiologist* OR radiographer* OR clinician* OR physician* OR (asthma ADJ10 inhal*) OR pathology | intervent* OR behavio* OR incentiv* OR strateg* OR messaging OR educat* OR program* OR training OR implement* OR communicat* OR nudg* OR choice* OR e-nudge OR "decision support" OR audit OR feedback |
|  |  |  |
| **CINAHL** |  |  |
| (emission* N10 (environment* OR "greenhouse gas*" OR carbon OR gas OR gases OR pollut* OR CO2)) OR "carbon footprint" OR "greenhouse gas" OR "greenhouse gases" OR (environment* N1 impact*) OR "climate change" OR "climate friendly" OR "climate-friendly" OR "CO2e"OR "carbon dioxide equivalents" | doctor OR doctors OR nurse OR nursing OR nurses OR dentist* OR an#esthe* OR "health professional*" OR surgery OR surgeries OR surgical OR (health OR hospital N3 (staff OR provider)) OR (imaging N3 (medical OR nuclear OR diagnostic OR radiological OR ultrasound)) OR "magnetic resonance imaging" OR MRI OR "computed tomography" OR radiograph* OR radiologist* OR radiographer* OR clinician* OR physician* OR (asthma N10 inhal*) OR pathology | intervent* OR behavio* OR incentiv* OR strateg* OR messaging OR educat* OR program* OR training OR implement* OR communicat* OR nudg* OR choice* OR e-nudge OR "decision support" OR audit OR feedback |
|  |  |  |
| **WEB OF SCIENCE/PROQUEST** |  |  |
| (emission* NEAR/10 (environment* OR "greenhouse gas*" OR carbon OR gas or gases OR pollut* OR CO2)) OR "carbon footprint" OR "greenhouse gas" OR "greenhouse gases" OR (environment* NEAR/1 impact*) OR "climate change" OR "climate friendly" OR "climate-friendly" OR "CO2e" OR "carbon dioxide equivalents" | doctor OR doctors OR nurse OR nursing OR nurses OR dentist* OR an*esthe* OR "health professional*" OR surgery OR surgeries OR surgical OR ((health OR hospital) NEAR/3 (staff OR provider)) OR (imaging NEAR/3 (medical OR nuclear OR diagnostic OR radiological OR ultrasound)) OR "magnetic resonance imaging" OR MRI OR "computed tomography" OR radiograph* OR radiologist* OR radiographer* OR clinician* OR physician* OR (asthma NEAR/10 inhal*) OR pathology | intervent* OR behavio* OR incentiv* OR strateg* OR messaging OR educat* OR program* OR training OR implement* OR communicat* OR nudg* OR choice* OR e-nudge OR "decision support" OR audit OR feedback |

**Appendix 2 – Behaviour change techniques - full codes from papers**

| **Author (year)** | **Behaviour Change Techniques (BCTs)** | | | | | | | | | | | | | Total number of BCTs used |
| --- | --- | --- | --- | --- | --- | --- | --- | --- | --- | --- | --- | --- | --- | --- |
|  | 1.3 | 1.6 | 2.1 | 2.7 | 3.1 | 4.1 | 5.2 | 5.3 | 7.1 | 9.1 | 10.8 | 12.1 | 12.5 |  |
|  | Goal setting (outcome) | Discrepancy between current behaviour and goal | Monitoring of behaviour by others without feedback | Feedback on outcome(s) of behaviour | Social support (unspecified) | Instruction on how to perform a behaviour | Salience of consequences | Information about social and environmental consequences | Prompts / cues | Credible source | Incentive (outcome) | Restructuring the physical environment | Adding objects to the environment |  |
| Epstein et al, 2016 |  | Percent deviations from target FGF values were provided along with a reminder of the desired FGF for each agent |  | Every fourth Monday an email was sent to every provider listing the average intraoperative FGF for their most recent 10 cases with each volatile agent |  | Email to providers encouraging them to maintain 1L/min FGF during the maintenance of sevoflurane administration instead of increasing the FGF to 2L/min |  |  | Notified by email. Added a notification to change the absorbent to the text message to turn over the operating room. Added an alert to the technicians to check the absorbent | Change in policy was approved and endorsed by the chair and vice chair of the department |  | Canisters containing exhausted soda lime were refilled with the nonreactive absorbent | Place a note on the anaesthesia machine requesting the absorbent to be changed | 7 |
| Regan et al, 2018 | Target reduction |  |  | Initial results were communicated to the team. Illustrating the financial and carbon savings achieved to date | A progress report was published and communicated to the ward team thanking them for a positive engagement with the project | Educational poster provided a practical guide to selecting the common C005 test required on the computer system | Cartoon-based stickers. | The poster illustrated the issues of resource use within the NHS and the wider environmental implications of wasteful practices | Stickers fixed onto computer screens. Posters. Progress reports | Intervention created by the department | Incentive of a celebratory tea trolley for staff if the reduction target was met and maintained |  | Educational poster placed throughout the ward in clinical and rest areas and cartoon based stickers fixed onto the computer screens | 10 |
| Carter et al, 2019 |  |  |  | Feedback on actual costing figures based on information from pharmacy about monthly spending. Monthly spot audits of gas flow rates. Feedback on the latest spot audit findings at weekly anaesthetic departmental meetings | Email updates highlighting improvements we had made as a group to encourage continued engagement | Representative from the department to advise as to the optimal use of desflurane | 'Low flow board' highlighted project aims alongside pictorial and graphical representation of monthly progression | Presented these data together with their environmental and economic impact at our weekly departmental meeting | Regular email updates to the anaesthetic body | Support from departmental lead clinician |  | Making isoflurane vaporisers available in all anaesthetic rooms and inside theatres and removing sevoflurane vaporisers from inside all theatres | Low flow board' placed on the wall in the anaesthetic department | 9 |
| Zuegge et al, 2019 |  |  |  |  | Efforts were made via outreach relationship building and one-on-one conversations…addressing any concerns as well as empowering local passionate champions | Provider information regarding volatile agent waste was incorporated into the annual waste reduction and green practices lecture. A specific new employee training presentation was developed | A graphic designer was employed to create labels with images intended to elicit an emotional response | A specific new employee training presentation was developed that included volatile agent waste and environmental harm | Email communications distributed every few months. Labels added | Intervention created by the department |  |  | Vaporiser labels were developed to guide providers at the point of decision making | 7 |
| Glenski et al, 2020 |  | The low flow wizard displays the required FGF and the user's current flows | Confirmation rounds were performed on a random basis. This involved checking to see whether the provider was utilising the low flow wizard | Every Friday an update would occur regarding the confirmation rounds, the decrease in sevoflurane used per anaesthetic and the environmental impact of the project | Gather around the huddle board each morning. Forum to answer questions, educate and present project progress | Multiple educational items were presented to all anaesthesia providers in the department which included how to use the low flow wizard on our machines | Translate this to carbon footprint equivalents of miles driven by an average car, gallons of gasoline and pounds of coal burned | The many benefits of low flow anaesthesia including decreased costs and environmental impact were also distributed | If the FGFs are turned down too low, the low flow wizard will notify the user that they need to be increased | Department of anaesthesiology officially adopted this QI initiative |  | The low flow wizard was configured to be on the default screen of all our machines |  | 10 |
| McAlister et al, 2021 |  |  |  |  | Meetings, staff orientations | Poster telling doctors when to order tests | Slogans like 'more is not always better' |  |  | Developed in conjunction with heads of department |  |  | Strategic placement of posters in areas frequented by junior doctors | 5 |

**Appendix 3 – Behaviour change techniques - full codes from abstracts**

| Author (year) | Behaviour Change Techniques | | | | | | | | |
| --- | --- | --- | --- | --- | --- | --- | --- | --- | --- |
|  | 1.3 | 2.7 | 4.1 | 5.2 | 5.3 | 7.1 | 9.1 | 12.1 | 12.5 |
|  | Goal setting (outcome) | Feedback on outcome(s) of behavior | Instruction on how to perform a behaviour | Salience of consequences | Information about social and environmental consequences | Prompts / cues | Credible source | Restructuring the physical environment | Adding objects to the environment |
| Patel et al, 2014 | Goal to reduce mean FGF for ISO and DES to 1.0 l/min and SEV to 2.0 l/min | Each anesthesia provider was emailed a report describing his or her FGF for each agent over the prior 12 months |  |  | Presented the program as an environmental initiative | Individualized reports for each agent over the previous 12 weeks were emailed on Jul 1, Sep 4, Sep 23, and Oct 21, 2013 | Initiative from the department |  |  |
| Boyle et al, 2018 |  |  |  |  | Talks were given to theatre staff about the environmental impact of volatile agents |  | Initiative from the department | Default agents on our machines would be sevoflurane and isoflurane, and desflurane would be available on a request basis only | Purchase of new anaesthetic machines |
| Danby et al, 2018 |  | Data collected was presented to the department |  | Results were converted to emission data for a Golf GTI (148 gCO2.km). results were equated to the total distance travelled in the car. | Highlighted the environmental impact of volatile use with an emphasis on equivalent distances travelled by the car |  | Initiative from the department |  |  |
| Jani & Kalla, 2018 |  | Findings were shared with colleagues and trainees within the department | A reminder to switch off the gases on transfer and to reduce the flow during anaesthesia |  |  | Visual prompts as a reminder to switch off the gases on transfer and to reduce the flow during anaesthesia. email reminders. | Initiative from the department |  | Displayed visual prompts on the theatre doors and the anaesthetic machine in anaesthetic rooms, as well as theatres |
| Hickman & Molyneux, 2019 |  |  | EnVolwebsite, which provides a number of relevant educational resources. | Quantified this into tangible everyday equivalencies, such as coal burnt or miles driven | Prompt cards containing facts regarding volatiles and the environment were placed on top of all anaesthetic machines | Prompt cards containing facts regarding volatiles and the environment were placed on top of all anaesthetic machines | Initiative from the department |  | Prompt cards containing facts regarding volatiles and the environment were placed on top of all anaesthetic machines |
| Lawson & Baxter, 2019 |  |  |  |  |  |  | Initiative from the department | Desflurane was removed from all machines and the vaporiser setup was standardised to sevoflurane and isoflurane on every machine |  |
| Self & Eveleigh, 2019 |  |  | Suggested switching to sevoflurane or total intravenous anaesthesia. |  | Presentation was given to the anaesthetic department at the Trust highlighting recent publications on the environmental impact of desflurane |  | Initiative from the department |  |  |
| Benness & Doane, 2021 |  |  | Encouraged alternatives to desflurane |  | Departmental presentation. Geared at staff education and encouraged alternatives to desflurane | Departmental newsletters | Initiative from the department |  | Placement of educational posters across targeted areas |
| Carta et al., 2021 |  | Email was circulated with the audit results and recommendations | Results were presented at the clinical governance meeting in June 2020 for which anaesthetists were encouraged to use low flows during general anaesthesia along with increased use of sevoflurane | Indicating the CO2 equivalence per hour of each volatile agent along with the distance equivalent (in kilometres) in a motor vehicle. | Indicating the CO2 equivalence per hour of each volatile agent | Stickers were placed on all anaesthetic machines | Initiative from the department |  | Stickers were placed on all anaesthetic machines |
| Hirst et al., 2021 |  |  |  |  |  |  | Initiative from the department | Desflurane vaporisers were removed from anaesthetic machines |  |
| Jameson & Young, 2021 | Objective was for 95% of general anaesthetic cases to achieve end tidal control within 1 min of securing the airway. |  | Educational presentation |  |  | Email reminders | Initiative from the department |  |  |
| Kirkman et al., 2021 |  |  |  |  | Presented at the monthly clinical governance meeting...the volatile supplied to theatres was converted to CO2equivalents. |  | Initiative from the department | Desflurane was removed as a default vaporiser on all anaesthetic machines | Educational posters were displayed in anaesthetic rooms |
| Roome et al, 2021 |  |  | A summary of greener inhalers was distributed to all prescribers |  |  | Prompt appears when Ventolin in prescribed to recommend Salamol instead | CCG made reducing theuse of high carbon inhalers a key prescribing priority |  |  |
| Wilson & Clark, 2021 |  | Volatile anaesthetic agent usage was presented to the department |  |  |  |  | Initiative from the department |  |  |

**Appendix 4 – Risk of bias information**


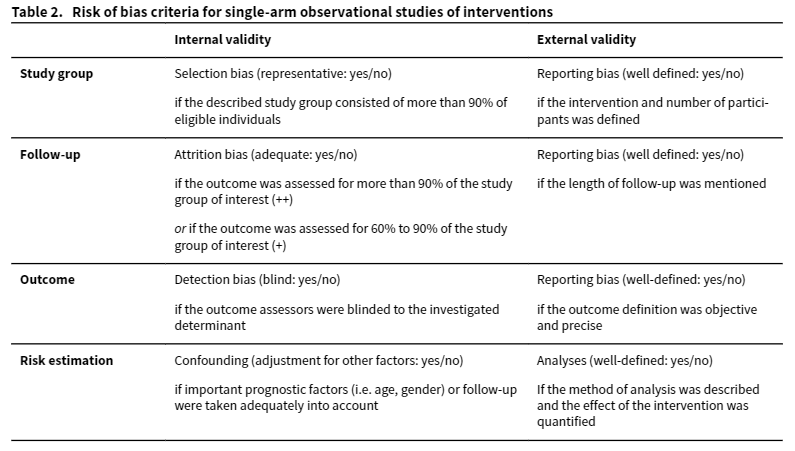


**Appendix 5 – Risk of bias - more detailed table**

|  | Selection bias? - no selection bias means sample representative of sample planned to be studied | | Attrition bias? (yes/no/unclear) | | Detection bias? (yes/no/unclear) | | Confounding? - is there likely to be confounding (potential for bias: yes/no/unclear) | | Is the study group likely to be generalisable? (potential for bias yes/no/unclear) | | Was the duration of follow-up adequate/appropriate for the question? (potential for bias yes/no/unclear) | | Was the outcome well defined and appropriate (potential for bias: yes/no/unclear) Minimal environmental important difference (MEID)? | | Are the analyses appropriate (potential for bias: yes/no/unclear) | |
| --- | --- | --- | --- | --- | --- | --- | --- | --- | --- | --- | --- | --- | --- | --- | --- | --- |
|  | Judgement | Rationale | Judgement | Rationale | Judgement | Rationale | Judgement | Rationale | Judgement | Rationale | Judgement | Rationale | Judgement | Rationale | Judgement | Rationale |
| Epstein et al., 2016 | No | Study group has all eligible individuals | No | Outcomes assessed for whole department | Yes | Assessors involved in intervention | Unclear | Aimed to capture confounding variables but did not | Unclear | Did not define number of people in department (in intervention) | No | Defined follow up adequately | No | Defined outcome adequately | No | Defined method of analysis and quantified effect of intervention |
| Regan et al., 2018 | No | Study group has all eligible individuals | No | Outcomes assessed for whole department | Yes | Assessors involved in intervention | Aimed to capture confounding variables but did not | Did not control for any confounders | Unclear | Did not define number of people in department (in intervention) | No | Defined follow up adequately | No | Defined outcome adequately | Yes | Did not perform any statistical analyses |
| Carter et al., 2019 | No | Study group has all eligible individuals | No | Outcomes assessed for whole department | Yes | Assessors involved in intervention | Yes | Did not control for any confounders | Yes | Did not define number of participants or intervention adequately | No | Defined follow up adequately | No | Defined outcome adequately | Yes | Did not perform any statistical analyses |
| Zuegge et al., 2019 | No | Study group has all eligible individuals | No | Outcomes assessed for whole department | Yes | Assessors involved in intervention | Yes | Did not control for any confounders | Yes | Did not define number of participants or intervention adequately | No | Defined follow up adequately | No | Defined outcome adequately | No | Defined method of analysis and quantified effect of intervention |
| Glenski & Levine, 2020 | No | Study group has all eligible individuals | No | Outcomes assessed for whole department | Yes | Assessors involved in intervention | Yes | Did not control for any confounders | Unclear | Did not define intervention adequately | No | Defined follow up adequately | No | Defined outcome adequately | No | Defined method of analysis and quantified effect of intervention |
| McAlister et al., 2021 | No | Study group has all eligible individuals | No | Outcomes assessed for whole department | Yes | Not blinded to group | Unclear | Aimed to capture confounding variables but did not | Unclear | Did not define number of people in department (in intervention) | No | Defined follow up adequately | No | Defined outcome adequately | No | Defined method of analysis and quantified effect of intervention |

**Appendix 6 – TiDIER table**

|  | **Brief name** | **Why?** | **What?** | **Who provided?** | **How?** | **Where?** | **When and how much?** | **Tailoring** | **Modification** | **How well?** |
| --- | --- | --- | --- | --- | --- | --- | --- | --- | --- | --- |
| **Epstein et al., 2016** | An intervention to reduce fresh gas flow | To change cost and reduce wastage | Change in policy Regular email updates Public announcements Personalised feedback  Canisters changed Reminder notes on machines | Anaesthesia providers (anaesthesiologists, nurse anaesthetists, anaesthesiology residents) within a hospital setting | As a group - email and face to face announcements at team meetings. Personalised email reports sent throughout study period | In a hospital setting. Grand Rounds meetings, strategic placing of reminders in the physical environment | One off intervention in May 2014. Measured before and after over 8 4 week periods | Both general and personalised. Every provider received the same prompts and announcements, but personalised emails were sent about individual usage | The anaesthetists were having difficulty knowing when to change the absorbent. So introduced text message notifications, physical prompts on the machines, and email reminders | Not assessed |
| **Regan et al., 2018** | An intervention to reduce combined biochemical tests ordered by half | To reduce financial costs and carbon dioxide emissions | Target set Educational posters Cartoon-based stickers Incentivised celebratory tea trolley for targets met | Clinical staff on ward who order biochemical tests | Three progressive rounds of educational posters and cartoon-based stickers were placed on computer screens on the ward. Round 2 provided initial results and an incentive for meeting the target. Round 3 provided final results | Paediatric Cardiology Ward (Bear Ward) in Great Ormond Street Hospital, London | 8-week educational intervention in 2015, with 3 rounds of intervention. | No tailoring | No modifications | Not assessed |
| **Carter et al., 2019** | An intervention to promote low-flow anaesthesia and encourage a more environmentally friendly gas (isoflurane instead of sevoflurane) | To make anaesthtic practice more environmentally friendly, and reduce department spending | Weekly team feedback Staff presentations Regular email updates Made isoflurane vaporisers available Removed sevoflurane vaporisers Poster marking progression | All anaesthetic consultants and trainees | Cycle 1 - presentation at staff meeting Cycle 2 - email updates Cycle 3 - introcution of an educational 'low flow' board in the department + made isoflurane vaporisers available in all theatres Cycle 4 - removed sevoflurane from the machines in the theatre Cycle 5 - presentation to new trainees | Royal Free Hospital anaesthetic department, North London | Five one-to-two-month cycles - September 2016-March 2017 | No tailoring | The intervention was modified at each cycle in line with progressive data input and hypothesising about the causes of noted effects. For example, after cycle 2 they noted that emails seemed less effective at driving the intended behaviour modification, so a physical board was implemented instead (cycle 3). Also, new trainees in the dept were observed so they presented to them in cycle 5. | Not assessed |
| **Zuegge et al., 2019** | An intervention to reduce flor rates and volatile agent choice | To reduce costs and greenhouse gas emissions | Education: lectures and presentations Regular email updates  Labels added to machines Community support | A mulidisciplinary operating room team (including members from anaesthesiology, nursing, surgical technology, environmental services, information technology, and pharmacy) within a hospital | As a group - face to face education, and emails. Personlised feedback if they had concerns | In a hospital setting. Physical reminders, lectures | Usage of anaesthetic agents was tracked over 5 years | No tailoring | No modifications | Not assessed |
| **Glenski et al., 2020** | An intervention to increase use of low-flow anaesthetic | To reduce costs and emisssions | New machines Spot checks Weekly team feedback Education: demonstration and presentations New defaults set | Anaesthesia providers at Children's Mercy Hospital | All interventions delivered as a group. Both face-to-face e.g. meetings and demonstrations, and written information | In a hospital setting (Department of Anaesthesiology). Daily huddle board meetings, reminders in the physical environment | Implemented over 9 months. Measured the 12 month period before and after | No tailoring | No modifications | Sent a survey out to the whole department at the end of the intervention period. It asked “over the past year have you changed your practice and performed more TIVAs in an attempt to conserve sevoflurane use?” Only one anesthesiologist reported that they had increased the number of TIVAs over the last year, but did mention that it was “not necessarily to save Sevo.” |
| **McAlister et al., 2021** | An policy intervention to reduce non-urgent pathology testing | Policy aimed to reduce patient harm and economic costs; study aimed to see whether the policy change impacted on carbon emissions | Policy change Educational posters Department meetings Staff orientations | Division of Medicine at St George hospital | Posters in physical spaces for everyone. Department meetings and staff orientations face to face and in a group | In a hospital setting (Department of Medicine) - anywhere a patient was admitted for 24 hours or longer | Intervention took place September 2019 to February 2020 - ongoing | No tailoring | No modifications | Not assessed |
